# Relational autonomy: what does it mean and how is it used in end-of-life care? A systematic review of argument-based ethics literature

**DOI:** 10.1186/s12910-019-0417-3

**Published:** 2019-10-26

**Authors:** Carlos Gómez-Vírseda, Yves de Maeseneer, Chris Gastmans

**Affiliations:** 1 Interfaculty Centre for Biomedical Ethics and Law, KU Leuven, Kapucijnenvoer 35/3, B-3000 Leuven, Belgium; 2 Faculty of Theology and Religious Studies (Theological and Comparative Ethics), KU Leuven, Sint-Michielsstraat 4 - box 3101, B-3000 Leuven, Belgium

**Keywords:** Relational autonomy, End of life, Medical ethics, Review, Respect for autonomy, Decision making

## Abstract

**Background:**

Respect for autonomy is a key concept in contemporary bioethics and end-of-life ethics in particular. Despite this status, an individualistic interpretation of autonomy is being challenged from the perspective of different theoretical traditions. Many authors claim that the principle of respect for autonomy needs to be reconceptualised starting from a relational viewpoint. Along these lines, the notion of relational autonomy is attracting increasing attention in medical ethics. Yet, others argue that relational autonomy needs further clarification in order to be adequately operationalised for medical practice. To this end, we examined the meaning, foundations, and uses of relational autonomy in the specific literature of end-of-life care ethics.

**Methods:**

Using PRESS and PRISMA procedures, we conducted a systematic review of argument-based ethics publications in 8 major databases of biomedical, philosophy, and theology literature that focused on relational autonomy in end-of-life care. Full articles were screened. All included articles were critically appraised, and a synthesis was produced.

**Results:**

Fifty publications met our inclusion criteria. Twenty-eight articles were published in the last 5 years; publications were originating from 18 different countries. Results are organized according to: (a) an individualistic interpretation of autonomy; (b) critiques of this individualistic interpretation of autonomy; (c) relational autonomy as theoretically conceptualised; (d) relational autonomy as applied to clinical practice and moral judgment in end-of-life situations.

**Conclusions:**

Three main conclusions were reached. First, literature on relational autonomy tends to be more a ‘reaction against’ an individualistic interpretation of autonomy rather than be a positive concept itself. Dichotomic thinking can be overcome by a deeper development of the philosophical foundations of autonomy. Second, relational autonomy is a rich and complex concept, formulated in complementary ways from different philosophical sources. New dialogue among traditionally divergent standpoints will clarify the meaning. Third, our analysis stresses the need for dialogical developments in decision making in end-of-life situations. Integration of these three elements will likely lead to a clearer conceptualisation of relational autonomy in end-of-life care ethics. This should in turn lead to better decision-making in real-life situations.

## Background

Respect for autonomy has become a key concept in contemporary bioethics. The Belmont Report originally conceptualised respect for autonomy under the notion of respect for persons [1]. Beauchamp and Childress further developed and popularised the concept in the book *Principles of Biomedical Ethics* [2]. Since then, respect for autonomy, along with the other principles of beneficence, nonmaleficence, and justice, has provided the dominant theoretical framework for medical ethics. Nonetheless, the nature and value of this principle still generates much debate. An individualistic understanding of autonomy has been criticized from different theoretical standpoints. These criticisms have resulted in significant philosophical works calling for a reconceptualisation of autonomy in relational terms [3–5].

In the same vein, authors of empirical ethics studies have argued that autonomy would be better understood from a relational perspective [6–9]. Especially for end-of-life ethics, the need for a relational turn in the understanding of autonomy has led to a growing number of publications on shared decision-making [10–13] and advance care planning [14–16]. Both of these practices in end-of-life care are unsatisfactorily conceptualised in the classical individualistic framework. Relational autonomy emerges here as an interesting concept for these trends in end-of-life decision-making. Empirical research about relational autonomy in this field highlighted potential changes in the doctor-patient relationship and in physicians’ responsibility towards patients and their families [17].

A trend of recent publications in ethical literature reveals a growing awareness of the relational dimension of healthcare, in general, and in end-of-life care practices, in particular. This heightened awareness suggests that a ‘relational turn’ in current thinking on autonomy is actually taking place. Nevertheless, much misunderstanding exists about relational autonomy, since it is used as an ‘umbrella term’, covering a range of diverse perspectives [3]. A review of argument-based literature concerning relational autonomy in end-of-life care ethics is lacking, to the best of our knowledge. Thus, the aim of this review was to clarify the meaning, foundations, and uses of the concept of relational autonomy in end-of-life care ethics through a systematic review of argument-based literature.

## Methods

Recently, it has been stated that modern scholarly development in biomedical ethics requires the conduct of systematic reviews [18]. The increasing use of this form of literature appraisal has led to different models of systematic reviews [18, 19]. Among these models are systematic reviews of argument-based literature, which aim to present up-to-date comprehensive overviews of the ethical arguments and underpinning concepts identified in relation to a certain topic [18]. This approach is important for acquiring objective evidence for better decision-making in the delivery of healthcare, development of policy, and conduct of medical research.

We performed a systematic review of argument-based literature in order to better understand the meanings, foundations, and uses of relational autonomy in the field of end-of-life care ethics. Firstly, we formulated our research questions; secondly, we conducted a systematic literature search using standard methods; thirdly, we identified and described the different meanings, foundations, and uses of relational autonomy.

### Research questions

To the best of our knowledge, no published reviews exist that specifically focus on the use of the concept of relational autonomy in end-of-life ethics. This prompted us to formulate the following research questions:

1. What is the meaning of relational autonomy in end-of-life care ethics?
2. What are the philosophical foundations of relational autonomy in end-of-life care ethics?
3. How is relational autonomy used in argumentations regarding end-of-life care ethics?

### Literature search

The research questions were distilled into three groups of concepts (Table 1) in order to organize our literature search. The purpose of Group A concepts was to focus on the use of relational autonomy through technical concepts and common expressions. Group B focused the search on end-of-life topics. Group C concepts constrained our search of publications to the ethics domain. Each group was then operationally expressed in specific database search terms in a suitable format for the different databases queries (Table 2).

**Table 1:** Groups of organising concepts and associated database search terms

A. Relational Autonomy	B. End of Life	C. Ethics
relational; relational autonomy; shared autonomy; family autonomy; relational approach; relational responsibility; relational care; relational anthropology; relational personhood; relational account; relational turn; relational dignity; human relatedness; interdependency; shared decision making; social autonomy; solidarity; communitarian; beyond autonomy; beyond individualism	euthanasia; palliative care; withholding treatment; terminal care; assisted suicide; pain management; right to die; death with dignity; hospice care; advance directives; attitude to death; resuscitation orders; advance care planning; euthanize; palliative sedation; do not resuscitate; do not intubate; supportive care; physician-assisted suicide; assisted death; aid in dying; medical assistance in dying; mercy killing; end of life; terminal therapy; patient comfort; comfort care; living will; dignity therapy; palliative therapy; treatment withdrawal	ethics; philosophy; bioethics; morals; ethical analysis; principle-based ethics; medical philosophy; medical ethics; theology; morality

**Table 2:** PubMed search strings stratified according to organising concepts*

Group A: Relational Autonomy		Group B: End of Life		Group C: Ethics
(relational [Title/Abstract] OR relational autonomy [Title/Abstract] OR shared autonomy [Title/Abstract] OR social autonomy [Title/Abstract]) OR family autonomy [Title/Abstract] OR relational approach [Title/Abstract] OR relational responsibility [Title/Abstract] OR relational care [Title/Abstract] OR relational anthropology [Title/Abstract] OR relational personhood [Title/Abstract] OR relational account [Title/Abstract] OR relational turn [Title/Abstract] OR relational dignity [Title, Abstract] OR human relatedness [Title/Abstract] OR interdependency [Title/Abstract] OR shared decision making [Title/Abstract] OR solidarity [Title/Abstract] OR communitarian*[Title/Abstract]) OR beyond autonomy [Title/Abstract] OR beyond individualism [Title/Abstract])	AND	(“euthanasia”[Mesh] OR “palliative care”[Mesh] OR “withholding treatment”[Mesh] OR “terminal care”[Mesh] OR “assisted suicide”[Mesh] OR “pain management”[Mesh] OR “death with dignity”[Mesh] OR “patient comfort”[Mesh] OR “hospice care”[Mesh] OR “advance directives”[Mesh] OR “attitude to death”[Mesh] OR “resuscitation orders”[Mesh] OR “advance care planning”[Mesh] OR “euthanasia”[Title/Abstract] OR “palliative care”[Title/Abstract] OR “withholding treatment”[Title/Abstract] OR “terminal care”[Title/Abstract] OR “assisted suicide”[Title/Abstract] OR “pain management”[Title/Abstract] OR “death with dignity”[Title/Abstract] OR “patient comfort”[Title/Abstract] OR “hospice care”[Title/Abstract] OR “advance directives”[Title/Abstract] OR “attitude to death”[Title/Abstract] OR “resuscitation orders”[Title/Abstract] OR “advance care planning”[Title/Abstract] OR euthanize*[Title/Abstract] OR “symptom control”[Title/Abstract] OR “palliative sedation”[Title/Abstract] OR advance directive*[Title/Abstract] OR do-not-resuscitate [Title/Abstract] OR “do not resuscitate order”[Title/Abstract] OR “do not intubate order”[Title/Abstract] OR “supportive care”[Title/Abstract] OR “physician-assisted suicide”[Title/Abstract] OR assisted-death*[Title/Abstract] OR “aid in dying”[Title/Abstract] OR “medical assistance in dying”[Title/Abstract] OR “mercy killing”[Title/Abstract] OR “end of life”[Title/Abstract] OR “terminal therapy”[Title/Abstract] OR “comfort care”[Title/Abstract] OR “right to die”[Title/Abstract] OR “living wills”[Title/Abstract] OR “dignity therapy”[Title/Abstract])	AND	(“ethics”[Mesh] OR “philosophy”[Mesh] OR “bioethics”[Mesh] OR “morals”[Mesh] OR “ethical analysis”[Mesh] OR “principle-based ethics”[Mesh] OR “medical philosophy”[Mesh] OR “medical ethics”[Mesh] OR “theology”[Mesh] OR ethic*[Title/Abstract] OR philosoph*[Title/Abstract] OR bioethics*[Title/Abstract] OR moral*[Title/Abstract] OR principle-based-ethic*[Title/Abstract] OR medical-philosoph*[Title/Abstract] OR medical-ethic*[Title/Abstract] OR theolog*[Title/Abstract])

*Other literature database search strings (not shown) were developed using *PubMed* strings as a template

Eight electronic literature databases were queried, which as a group covered the fields of healthcare sciences, philosophy, and theology. The eight databases were *PubMed, Embase, Web of Science, Scopus, Philosopher’s Index, Atla, Index Theologicus,* and *Index Religiosus*. All databases were queried using Boolean searches expressed in English. Table 3 presents the number of results returned using the search terms (and their complimentary terms used for the other database searches).

**Table 3:** Number of positive results (“hits”) in each database

Database	Search date	Number of results
*PubMed*	09/02/2019	464
*Embase*	09/02/2019	549
*Web of Science*	09/02/2019	783
*Scopus*	09/02/2019	510
*Philosopher’s Index*	09/02/2019	110
*Atla*	09/02/2019	15
*Index Religiosus*	09/02/2019	12
*Index Theologicus*	09/02/2019	3
	Total	2445

The search strings were developed by the first author (CGV) in consultation with the third author (CG). The final search strategies were reviewed and validated by an independent librarian from the KU Leuven Library.

The database search was performed in February 2019, using no filters or data restrictions. Resulting citations of the identified articles were managed in an EndNote (version X9, Clarivate Analytics, Philadelphia, PA, USA) reference library. Duplicate references were removed through the duplicate deletion function of the EndNote software.

The first author (CGV) successively screened titles, abstracts, and full texts of identified articles, following predefined inclusion and exclusion criteria (see below). In order to assess our criteria consistency, abstract screening was performed independently by the first (CGV) and third author (CG). In 96% of the abstracts (1145/1183), there was agreement about inclusion or exclusion. Both appraisers discussed every marginal or doubtful candidate article individually until consensus was reached.

To be included in the systematic review and appraisal, candidate articles had to meet all inclusion criteria and have no exclusionary one. Inclusion criteria were the following: (1) significant use of a relational approach to the concept of autonomy, (2) application to the specific field of end-of-life care. Using Broeckaert’s theoretical framework, we defined end-of-life care to include ‘any kind of treatment decisions that can be taken in advanced stages of life-threatening diseases’ [20]. These treatment decisions covered three main categories: curative or life-sustaining treatment, pain and symptom control, and euthanasia and assisted suicide. The third and fourth inclusion criteria were: (3) publication is considered to be in the argument-based literature, which is an article using ethical concepts derived from current or traditional ethical theories in order to argue for a position or conclusion [21]; (4) must be published in English, French, German, Portuguese, or Spanish.

We excluded editorials, book chapters, position papers, guidelines, reviews, protocols, ethics policies, and ethics codes.

Finally, we included any additional candidate publications that were undetected in the eight database searches but were later identified using the snowball method, citation tracking, or through personal experience with particular articles that were appropriate.

**Fig. 1:**
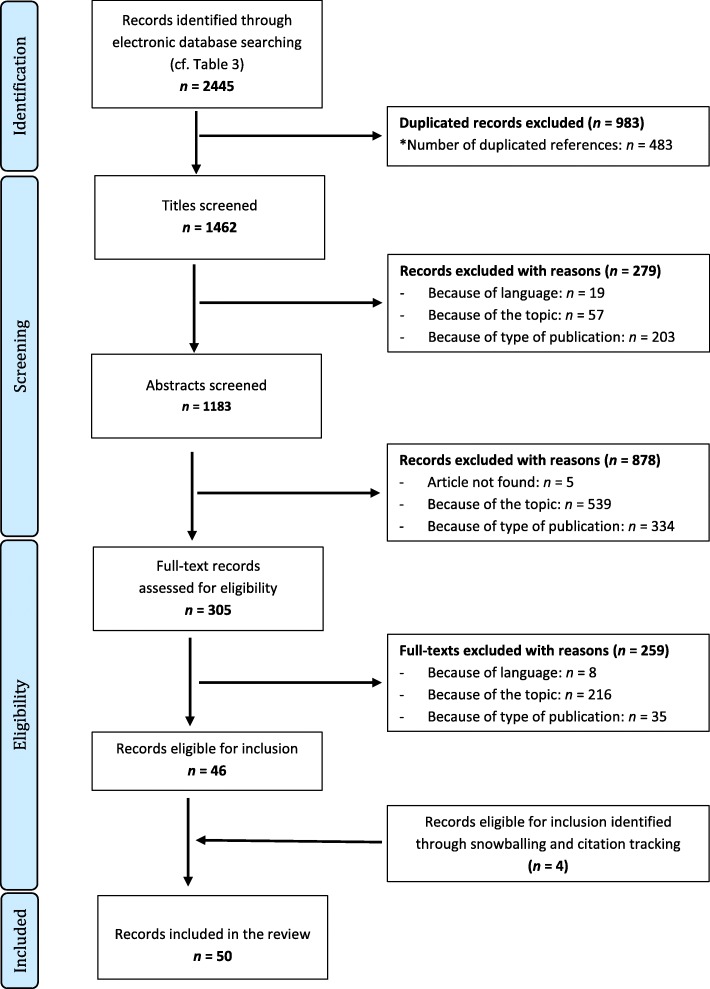
Flow chart showing the electronic search, identification, and selection process for the reviewed articles [23]

We followed the guidelines detailed in Peer Review of Electronic Search Strategies (PRESS) [22], and our search process and reporting followed the statement and flowchart of the Preferred Reporting Items for Systematic Reviews and Meta-Analyses (PRISMA) [23] (Fig. 1.).

### Data extraction and synthesis

For extraction and synthesis, we used the Qualitative Analysis Guide of Leuven (QUAGOL) approach, which consists of five preparatory, sequential steps [24]. First, articles were read and reread, highlighting the relevant parts and the main arguments presented. Second, we developed a narrative summary of these highlighted parts of the articles. The aim was to draw strong lines of argumentation and identify where in the article the main concepts appear. Third, a conceptual scheme for each publication was created. A conceptual scheme is a synthetic frame where different concepts that appear relevant to answer the research questions are presented and interrelated with each other (an example of a conceptual scheme is provided as Additional file 1). Each conceptual scheme was appraised separately by two authors (CGV and CG) so that we could objectively and accurately characterise each included publication. Both appraisers discussed the resulting conceptual schemes until they agreed on their adequateness. Fourth, these individual conceptual schemes were considered as a whole to search for relationships that would produce a comprehensive overall response to our research questions. Here, we aimed to focus on our research questions even if the individual article’s main concern was somewhat different from these issues. We built up a separate global scheme that integrated the most relevant meanings, foundations, and uses of the concept of relational autonomy. This scheme was iteratively evaluated and checked against previous QUAGOL steps in order to ensure that it was consistent. In the final and fifth step, we synthesised a description and report of these results to be presented in the Results section of this review.

## Results

Fifty articles met our inclusion criteria and were appraised for our research questions [25–74]. Their main characteristics are presented in Table 4. Publication dates ranged from 1999 to 2018, with 28 of them being published in the last 5 years. The great majority of the included articles were published in English (*n =* 42), although they originated from authors affiliated with institutions across a wide geographical spread. The most frequently represented countries were the USA (*n* = 10); Canada (*n* = 7); the UK (*n* = 6); and Belgium (*n* = 5).

**Table 4:** Description of characteristics of included publications

Analysed features	Number of Publications
Language	
English	42
German	3
French	2
Spanish	2
Portuguese	1
First authors’ publication country	
USA	10
Canada	7
UK	6
Belgium	5
Switzerland	4
Singapore	3
Australia	2
Germany	2
Ireland	2
Austria, Brazil, Chile, Cuba, France, Hong Kong, Netherlands, Poland, Spain	1 (*each country*)
Year of publication	
2015–2018	18
2010–2014	16
2005–2009	10
1999–2004	6
Ethical approach^a^	
Feminist ethics	21
Political philosophy	11
Care ethics	10
Ethical multiculturalism	8
Phenomenology	8
Personalist ethics	5
Relational ethics	4
Virtue ethics	1
End-of-life topics^a^	
Curative or life-sustaining treatment	32
Pain and symptom control	10
Euthanasia and assisted suicide	12

^a^Note: A single article can be represented more than once

**Fig. 2:**
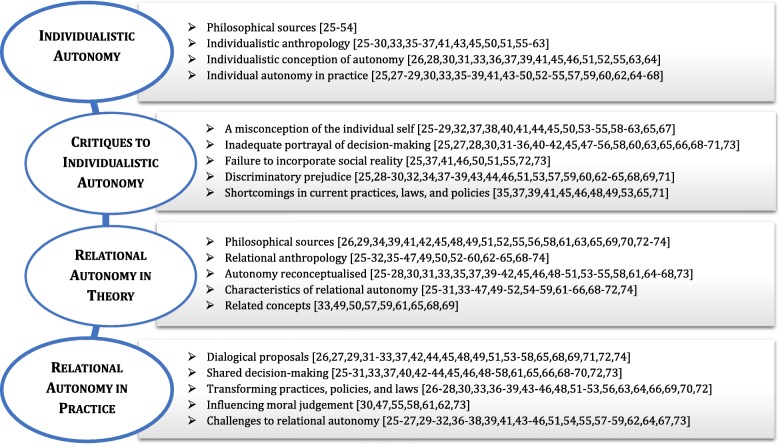
Fourfold global scheme emerging from analysis of the 50 included articles

As a result of the analysis and synthesis of the fifty individual articles, a fourfold structure was conceived by the authors (Fig. 2). The first two sections present and then criticize a simplified interpretation of individualistic autonomy, against which relational autonomy is often developed. These two preliminary steps are necessary in order to better apprehend the last two sections, where relational autonomy is elaborated in theory and in practice. In summary, we present our results in four main sections. First, we introduce a simplified interpretation of individual autonomy in mainstream bioethics, as derived from the analysis of the included publications. Second, we assemble critiques toward this individualistic interpretation. Third, relational autonomy is theoretically conceptualised using the adjusted understanding from the first two sections. Fourth, this new conceptualisation of relational autonomy is applied to scenarios of clinical practice and moral judgment in end-of-life situations.

### An individualistic portrayal of autonomy

Relational autonomy was often characterised by an oppositional response to the individualistic interpretation of autonomy. Thus, in many articles, authors started by portraying individual autonomy as a basic principle in end-of-life care ethics. On the basis of this supposition, the origins of an individualistic interpretation of autonomy were presented.

#### Philosophical sources

The philosophical origins of individual autonomy are temporally situated in the Modern era, in a thread that links the ideas of René Descartes [25–27], John Locke [28, 29], Immanuel Kant [25, 26, 29–35], and John Stuart Mill [26, 30, 31, 33, 36]. In contemporary bioethics, this line of thought finds expression in the notion of ‘respect for autonomy’, one of the four basic principles coined by Beauchamp and Childress in their monograph, *Principles of Biomedical Ethics* [2]. This classic book was referenced by 26 of the included publications [25, 26, 30–35, 37–54].

#### Individualistic anthropology

Any interpretation of autonomy is unavoidably underpinned by a certain view of what a human being is, in other words: by a particular philosophical anthropology. The anthropology derived from our analysis could be described in terms of self-determination [25, 27, 30, 33, 35, 37, 55–61]; independency [28, 29, 36, 41, 50, 51, 62]; self-awareness [26, 29, 43]; self-interest [25, 26, 29, 45]; and self-reliance [36, 45, 59]. Anchored by Christian and Western roots, the idea of personal identity, free will, and individual responsibility gave birth to a liberal conception of agency [26, 29, 33, 58, 63].

#### Individualistic conception of autonomy

In line with this individualistic understanding of human beings, autonomy is defined as ‘the ability to make individual, fully-informed, and independent decisions’ [28]. In this context, a large number of the publications discussed the conditions for an action to be considered autonomous. Firstly, the action has to be authentically intended [31, 39, 51]. Secondly, it has to be free from external interference of health professionals, relatives, or the society in general [28, 30, 33, 37, 39]. Thirdly, the agent has to be competent and sufficiently informed [26, 28, 36, 37, 41, 45, 46, 51, 52, 55, 63, 64].

#### Individual autonomy in practice

Authors acknowledged that respect for individual autonomy has served to protect patients against paternalism [27, 30, 36, 37, 39, 41, 43–45, 50, 52, 54, 59, 60, 62, 64, 65] and to help them overcome futile treatment decisions [37, 43, 47, 53]. Putting patients’ values, interests, and beliefs at the centre of healthcare decisions empowers them [29, 30, 33, 36, 45, 66, 67]. Application of the principle of autonomy in real-life situations has contributed to the development of patient’s rights, including privacy, confidentiality, self-determination, and primacy of truth-telling in end-of-life scenarios [25, 28, 33, 35, 37, 38, 44, 49, 54, 55, 57, 59, 62]. Included articles highlighted the point that the notion of individual autonomy is consistent with commonly used legal and ethical standards in end-of-life decision-making, namely informed consent [25, 27, 29, 30, 33, 35, 38, 47, 52]; advance directives [25, 27, 35, 37, 48, 49, 52, 53, 59, 60, 65, 68]; surrogate decision making [35, 37, 46, 48, 53, 65]; and the best-interest standard [37–39, 45–47, 64, 65].

### Critiques of an individualistic interpretation of autonomy

Although positive conceptualisations of individualistic autonomy have achieved much, these views are not unassailable. Respect for autonomy is widely accepted as a cornerstone in end-of-life care ethics, but mainstream interpretation of this idea has received many critiques as well. Critiques against an individualistic interpretation of autonomy cover five essential aspects. We consider them in turn.

#### Misconception of the individual self

Authors advocating a relational approach to autonomy argued against an individualistic portrayal of autonomy as a misconception of the individual self [25, 27–29, 37, 38, 41, 45, 55, 58–60, 62, 63]. For example, the individualistic portrayal promotes the ideas that the autonomous agent is supposed to be an atomistic self [29, 41, 44, 61, 62]; sovereign and unified [58, 62]; self-transparent to their individual beliefs and values [44, 53, 55, 65]; and self-interested in their strategic choices [25–27, 29, 37, 50, 53, 67]. It is not surprising, then, why some authors warned that this liberal picture is too abstract and fails to incorporate the social context [26, 32, 54]. This is particularly important for end-of-life care, which Marx and colleagues characterised as a ‘relational process’ [40].

#### Inadequate portrayal of decision-making

According to some critics, common discussions about decision-making tend to consider generic patients in idealised circumstances [52, 53, 55]. However, in the case of serious illness, the circumstance is usually a very physical and emotionally demanding experience, which affects one’s ability to choose [25, 33, 50, 58, 66, 69]. In fact, authors adopting a relational approach to autonomy referred to empirical studies showing that severe illness dampens patients’ preferences for active participatory roles [53, 66].

Canonical discussions about individual autonomy interpreted it as an all-or-none affair [27, 28, 30, 31, 45, 51, 65]. Therefore, if the patient is declared free, competent, and authentic, the healthcare team must follow the patient’s decisions. If the patient lacks one of these three conditions, then somebody else takes on the role of decision-maker in the patient’s best interest. However, critics warned that this becomes problematic in many patients with fluctuating cognitive symptoms, or those who can be considered autonomous for certain actions but not for others [28, 52, 63, 65].

Another issue mentioned in many of the included publications was the misleading interpretation of the doctor-patient relationship. A Western perspective considers it to be a contractual relationship, one that can be thought of as a consumer-rights view of the patient [25, 35, 45, 55, 60, 63]. From this stance, the intrinsic asymmetry of the doctor-patient relationship is overlooked [25, 30, 33, 40, 69], and the importance of other values at stake, such as beneficence, care, responsibility, nonmaleficence, etc., go unnoticed [25, 34, 45, 47, 49, 56, 65, 70]. In particular, an individualistic understanding of autonomy seems to disregard important social values, such as justice, solidarity, and social responsibility [34, 41, 42, 47, 58, 71].

Two more false presuppositions were highlighted in the included articles that related to the aspect of an inadequate portrayal of decision-making. First, decision-making was better depicted as being a dynamic ongoing process rather than an isolated discrete event [25, 36, 69]. Second, decision-making was described as not being an exclusively rational act [28, 42, 45, 52, 54, 55, 63, 65, 68, 69]. Relational theorists have highlighted the importance of emotions, imagination, and non-verbal communication, as essential elements of human decision-making [27, 28, 32, 45, 48, 52, 54, 68, 69, 73].

#### Failure to incorporate social reality

The third aspect relates to a failure to incorporate social reality. The importance of particular relationships, such as family, friends, and communities, was commonly neglected by individualistic theories [25, 27, 32, 37]. Many authors insisted that end-of-life decisions *affect others* through many consequences [25, 28, 37, 41, 48–50, 53–55, 63], and *are affected by others’* concerns and opinions [25, 37, 41, 46, 50, 51, 55, 72, 73]. Rather than ideal models of self-sufficiency and independence, Wright stated that people’s way of decision-making at the end of life is *in consultation with* and *in consideration of* others [53]. Some authors lamented the alienating situation in which the decision-maker, either patient or surrogate, is isolated in order to protect them from external influence [26, 27, 45, 65].

#### Discriminatory prejudice

Relational ethicists are especially sensitive to discriminatory issues. Five authors addressed the problem of autonomy from a disability perspective [30, 39, 53, 62, 63]. They denounced the potentially ‘ableist’ ideology that is underpinned by a capacity-centred approach to autonomy. By the same token, a better understanding of the condition of patients with dementia have effectively provoked society to rethink ‘personal identity’ in situations in which psychological continuity, rationality, and independency are lacking [28, 37, 65]. Five of the included articles explicitly addressed this increasing reality of dementia and older adults and how it relates to autonomy [28, 37, 63, 68, 71].

In addition to discrimination, many authors denounced an ethnocentric bias in mainstream bioethics. They affirmed that an individualistic conception of autonomy is too closely linked with Western cultural values. This aspect neglects alternative ethnocultural values, such as family harmony, filial piety, and community fealty [25, 29, 32, 38, 43, 44, 46, 51, 57, 59, 64]. These values are essential in collectivist decision-making societies [25, 37, 38, 43, 51, 60, 63]. Especially for end-of-life situations, the importance of truth disclosure was commented on as a culturally sensitive matter [32, 37, 51, 59]. According to ethnocentrically sensitive authors, cultural awareness is crucial from a global ethics perspective [34, 37, 38, 43, 44, 68, 69]. Likewise, due to the increasingly global migration phenomena, societies are becoming progressively multicultural. Thus, a pluralistic ethics needs to be developed and refined further [25, 38, 57, 60, 64].

#### Shortcomings in current practices, laws, and policies

The final aspect relates to deficiencies in current practices, laws, and policies. Some authors of the included publications pointed out deficiencies in end-of-life decision-making practices linked to individualistic approaches to autonomy. First, Mackenzie and Rogers asserted that using only cognitive tests to assess mental capacity does not adequately capture the reality of many patients in end of-life situations [39]. Hence, when a patient is declared incompetent solely on the basis of cognitive test results, the current gold standard of advance directives [35, 48, 49, 65] and advance care planning [41, 48, 71] are not satisfactorily implemented. Reasons given for this failure were overemphasis of individual exercise of control, focus on legal documents that leads to procedural formalism, inappropriate priority of writing communication, and lack of applicability in conditions of uncertainty. Some authors expressed similar concerns about the silver standard of substitute or surrogate decision-making and the bronze standard of the best-interest principle [37, 45, 46, 48, 53, 65, 71]. The need for interpretative discussion in these latter practices demands a relational framework rather than an individualistic one [35, 49, 65].

### Relational autonomy in theory

We now present the conceptualisation of relational autonomy as described in the included publications.

#### Philosophical sources

We identified some specific ethical approaches while doing our analysis. A majority of the publications used feminist ethics approaches or primarily drew upon feminist sources (*n* = 21). Other approaches consisted of care ethics (*n* = 10); ethical multiculturalism (*n* = 8); phenomenology (*n* = 8); personalist ethics (*n* = 5); relational ethics (*n* = 4); and virtue ethics (*n* = 1). A significant proportion of articles used a political-philosophical approach (*n* = 11), such as communitarianism, liberalism, among others.

The philosophical sources used by some approaches became manifest. Feminist and care ethicists frequently referred to the works of Carol Gilligan [29, 41, 45, 55, 58, 63] and Joan Tronto [48, 52, 58, 63]. Those that espoused personalist approaches mainly looked to the works of Paul Ricoeur [42, 56, 58, 61, 65, 69, 70]; Martin Buber [29, 56, 70, 74]; and Emmanuel Levinas [29, 56, 69, 70]. On the other hand, those who framed their articles around relational ethics mentioned the works of Vangie Bergum and John Dossetor [52, 73]. Finally, for various philosophical reflections, different articles mentioned the works of Charles Taylor [29, 39, 51, 58, 65]; Martin Heidegger [26, 29, 72]; and Hans Jonas [34, 49].

#### Relational anthropology

Our synthesis describes a relational understanding of human beings in terms of connectedness [29, 37, 41, 43, 44, 53, 58–60] and interdependency [28, 32, 38, 58, 59, 63, 65]. Human beings are embedded in a web of interpersonal connections with others. Therefore, according to some articles, one’s personal interests are not only self-centred but ‘others-centred’ as well [25, 45, 52]. Some authors concluded that it is impossible to separate people from their social environment [41, 42, 45, 72, 73] or their culture [29, 31, 42, 44, 46, 55, 64]. These findings indicate that a relational anthropology is more sensitive to contextual and cultural mediations.

We found that authors insisted on the notion of an embodied self [26, 42, 45, 62, 69, 73], which entails vulnerability [25, 28, 30, 35, 36, 39, 40, 42, 47, 50, 52, 55, 58, 68–70, 73, 74] and dependency on others’ care [29, 36, 37, 45, 47, 49, 50, 52, 58, 64, 68]. These anthropological characteristics were essentially linked with other aspects, such as reciprocity [25, 43, 55, 63, 72, 74], responsibility [25, 42, 45, 47, 49, 54–56, 68, 70, 74], and collaboration [27, 43, 55].

A relational anthropology stresses self-transcendence [27, 47, 70, 72, 74]; dynamism [25, 42, 43, 45, 57]; and narrativity [41, 65, 71] of the self. Personal identity is constituted by a life story that takes part in on-going communities with common traditions and future expectations. Głos [28] and Rigaux [65] noted that a dynamic concept of the self is of paramount importance for patients suffering from dementia, who can have their identity restored through a history shared with others. Finally, a dynamic perspective entails a diachronic view of decision-making, not to be reduced to a static moment but instead as a process unfolding over time [25, 36, 39, 40, 45, 55, 57, 69].

#### Autonomy reconceptualised

Most of relational autonomy theorists do not completely reject the notion of autonomy; rather, they argue that the principle should be reconceptualised [3]. Nevertheless, our analysis did not find a consensus on the definition of relational autonomy. What we actually observed in some articles was a relational examination of the two dimensions of autonomy (i.e. self-determination and self-governing) and the classical three conditions of autonomy (i.e. freedom, competence, and authenticity) [31, 33, 39, 45, 51, 55, 67].

Relational autonomy aims to maintain the essential aspect of autonomy, namely control over one’s life, while at the same time, incorporate insights of a socially embedded notion [50]. Even among most of relational theorists, the balance of rights between the individual and the social was inclined towards the former. This became clear in the case of conflict between the individual patient and his or her entourage: Priority was given to the patient [30, 35, 46, 50, 51, 53–55, 64, 67]. Hence, whenever the family or healthcare professionals tried to overrule the patient’s autonomy, even when looking out for their best interests, authors considered this to be an example of unwarranted paternalism, pressure, coercion, or manipulation [50, 55, 73].

Nevertheless, several articles repeatedly stressed that others’ influence does not necessarily impede autonomy but can actually enhance it [25, 26, 28, 30, 42, 45, 50, 58, 64, 66, 73]. In other words, autonomy should not only be protected from unsolicited pressure but also should be actively promoted [27, 28, 31, 37, 40, 41, 45, 48, 50, 51, 58, 61, 68]. Family members and healthcare professionals could contribute to the development of the patient’s decision-making capacity [26, 30, 31, 50, 68]. This could be done by presenting new possibilities [45, 50]; giving emotional support [27, 28, 50]; removing social barriers [30]; or bridging gaps between the patient and the social environment [35, 49, 50, 64, 65].

#### Characteristics of relational autonomy

A relational understanding of autonomy considers the social reality of the individual in making decisions. It is, therefore, more particularistic and contextual [31, 33, 37, 39, 41, 43, 44, 46, 58, 64, 71, 72]. Along these lines, some authors were inclined to interpret relational autonomy in terms of inclusiveness [26, 29, 58, 62, 65], while others were sensitive to cultural diversity [25, 30, 37, 38, 50–52, 54, 59, 64, 65, 69]. For many authors, autonomy was a matter of degree [25, 27, 28, 31, 45, 51, 56, 65], rather than an all-or-nothing principle. They considered autonomy to be expressed along a continuum, one whose value can vary in the dynamic process of care [36, 39, 42, 44, 50, 51, 57, 69, 71]. Authors insisted that relational autonomy has to be balanced by other relational values, such as compassion [26, 51, 52, 63]; hope [33, 47]; trust [27, 35, 37, 38, 40, 42, 47, 54, 57, 66, 68]; empathy [25, 26, 45, 54, 65]; solidarity [28, 29, 34, 49, 56, 61, 63, 70, 74]; and responsibility [25, 34, 37, 41, 42, 45, 47, 49, 51, 54–56, 58, 68, 70, 71, 74].

Relational autonomy in the included publications was understood both *causally* [26, 28, 30, 31, 45, 58] and *constitutively* [25, 26, 28–30, 35, 37, 41, 44, 45, 58, 63, 65, 71, 72]. The former focusses on how ‘social relationships impede or enhance autonomy’; while the latter focusses ‘on the social constitution of the agent or the social nature of the capacity of autonomy itself’ [3]. Authors taking a feminist stance, such as Donchin, preferred to argue for a *strong conception* of relational autonomy. By doing so, she recognised ‘a social component built into the very meaning of autonomy’, rather than a *weak conception*, which ‘restricts the formative role of social relations to early development’ [55].

#### Related concepts

Our analysis of included publications revealed many notions closely aligned with the essence of what is called ‘relational autonomy’ in feminist and care ethics critiques, yet expressed using different terms. This was especially frequent among authors affiliated with non-Anglo-Saxon institutions. These related concepts were autonomy-in-relation [57, 59]; extended, assisted, and delegated autonomy [65]; preference autonomy [33]; second-order autonomy [50]; diminished and partial autonomy [69]; and autonomy in responsibility and in solidarity [49]. Finally, some articles employed more remote notions in order to express similar insights. For example, two articles authored by European bioethicists used the term *accompaniment*, to describe an association of autonomy and solidarity, both social values that promote and limit each other [61, 68].

### Relational autonomy in practice

When applied to end-of-life care practices, relational autonomy can be categorised into a great variety of care proposals. In this regard, following Broeckaert’s theoretical framework [20], we found that the majority of articles focussed on curative or life-sustaining treatment (*n* = 32). The remainder focussed on palliative care, pain, and symptom control (*n* = 10), or euthanasia and assisted suicide (*n* = 12).

#### Dialogical proposals

Most of the included publications proposed different types of dialogical proposals as the best way to implement relational autonomy in end-of-life decision-making [26, 27, 29, 31–33, 42, 44, 45, 55–57, 65, 69, 71, 72, 74]. Only Walker and Lovat [26] and Wilson et al. [33] explicitly based their theoretical foundations on Jürgen Habermas’ communication theory and dialogical ethics.

Although there was great diversity among the dialogical proposals described in the articles, they did share some common features. For example, in these proposals, dialogue included multiple participants [29, 54, 55, 65, 71] and had to be done in a timely manner [26, 31, 33, 48, 49, 51, 57, 58, 68, 69, 71]. Some authors highlighted that patients and relatives preferred oral communication [37, 48, 71], which was consistent with the notion that individualised dialogue has the advantage of responding more flexibly under uncertain circumstances [48, 54, 71]. Some articles described the potential benefits for patients, relatives, and clinicians [27, 53, 54]. For instance, relatives were relieved from the burden of making decisions alone when the patient was incompetent [48, 54]. Finally, many authors mentioned that multidisciplinary healthcare teams should also engage in dialogue [27, 33, 42–44, 56, 57].

#### Shared decision-making

Wallner concluded that shared decision-making has become the ethical gold standard in end-of-life decisions [54]. In six publications, this practice was explicitly based on a relational understanding of autonomy [26, 27, 29, 45, 48, 54]. Patients, relatives, and healthcare professionals were seen as cooperative ‘partners in the decision’ [27, 28, 40, 48, 55].

These views of shared decision-making revealed that the roles of the different stakeholders were reinterpreted. *Patients* were placed at the centre, emphasising that their best interest has to be actively sought through a respectful dialogue [26, 37, 68, 72]. *Relatives* were encouraged to participate in decision-making [25, 30, 40, 46, 51, 53, 57, 66, 69, 72]. Previously, three levels of family involvement have been described in end-of-life situations: (1) Family members take part in decision-making along with the patient; (2) the patient asks the family to control the decision-making process; (3) the family decides alone despite the patient’s wish to participate [75]. Some articles considered the first two levels of involvement to be valid expressions of relational autonomy [43, 44, 46], but the third level to be a case of ‘compromised autonomy’ [29, 46]. *Healthcare professionals¸* for their part, were said to have a certain responsibility towards the needs of the patient and the family [31, 45, 52, 54, 68, 73]. They should actively engage with the patient and with others having some sort of personal connection with the patient [26, 31, 33, 50–54, 65, 68, 73]. They were to act as facilitators of the decision-making process [26, 27, 50, 55, 57] and defend the best interests of the patient, according to their technical competence and expertise [27, 33, 40, 42, 45, 54, 66]. Finally, many articles stated that *society in general* also plays an important role in the development of values, such as dignity, responsibility, respect for the vulnerable, etc. [30, 42–44, 49, 51, 56, 58, 61, 65, 70].

#### Transforming practices, laws, and policies in end-of-life care

Some authors highlighted that current legal standards are aligned with an individualistic view of autonomy [30, 36, 45]. Gilbar and Miola suggested that Western legal systems are not sensitive enough to the needs of collective approaches [51]. Mackenzie and Rogers, for their part, detected contradictions between the cognitivist approach to autonomy in British law and its practical application, which demands implicit relational presuppositions [39]. Along the same lines, Wright proposed that some gentle prodding or ‘nudges’ are needed in order to modify existing defaults and transform family involvement into a more positive view [53].

A practical way of doing so is through the adaptation of standardised documents. Two examples were found in our articles: familial advance directives and community-based informed consent documents. A familial advance directive is a document ‘signed by the patient together with the family’ that ‘communicate [s] the wish of the family as a whole’ about the patient’s advance care planning and the dying process [37]. A community-based informed consent is a variation of the traditional informed consent document; it ‘considers the influence of relatives that is wanted and expected by some patients’ [27]. Other articles described similar proposals aimed at triggering early and inclusive discussions about end-of-life care [26, 37, 48, 56].

These suggestions are underpinned by different forms of moderate *familialism*, in which the family has the ‘default but not the absolute authority in the decision-making process’ [37]. In some articles, the family is regarded as a unit of care in itself [27, 38, 46, 72]. As highlighted by many of the authors, focussing attention specifically on the family is congruent with holistic palliative care philosophy [28, 33, 36, 52, 56, 63, 64, 66, 69, 70, 72].

Some authors proposed new forms of end-of-life decision-making. Krishna and colleagues introduced the ‘welfare approach’, a model in which a multidisciplinary team makes the final decision about a patient’s end of life after considering the patient’s best interests and the relational context [43, 44]. ‘Instilled with local beliefs, values and experiences’, this model ‘aims to allow patients to enjoy autonomy as long as the decisions do not result in a negative outcome for their overall welfare’ [43]. Dudzinski and Shannon proposed the ‘negotiated reliance response’ [36]. In this model, caregivers try to maintain balance between respect for the vulnerable patient and respect for the patient’s autonomy. Concretely, this model may permit a caregiver to invade the patient’s privacy, for example, in order to achieve a shared and negotiated total good. Finally, Głos proposed the ‘supportive care approach’ [28]. This approach is based on cooperative solidarity between patients, carers, and the state, in order to collectively bear costs and burdens of the care of elderly patients at the end of life.

#### Influencing moral judgement in issues of end-of-life

Relational autonomy is sometimes used as a specific framework to analyse ethical issues at the end of life. In particular, we found that it is used as narrow lens to view aspects of medically assisted death [30, 47, 58, 61, 62, 73] or euthanasia [47, 56, 61]. Generally, authors writing about these topics react against an individualistic interpretation of a patient’s right to make voluntary decisions about their own life and death [30, 55, 62, 73]. They also point out the social and political elements at stake [30, 55, 58, 73]. Positions in favour of and against medically assisted death and euthanasia could be found throughout the included articles.

#### Challenges to applying relational autonomy in end-of-life practices

Publications also addressed many practical challenges when applying relational autonomy to end-of-life care ethics. The main concern was how to protect the patient against abuses and unwarranted interventions of family members [29, 31, 37, 43, 45, 51, 64, 67]. Some authors pointed out that futile treatment and therapeutic obstinacy may result from collective pressure [27, 43, 44]. Similarly, authors analysed the problem of paternalistic interventions coming from healthcare professionals [30, 31, 36, 41, 43, 44, 54, 62, 64]. In practice, doctor collusion and ‘silence conspiracy’ seemed to be practices more likely to happen in collectivist contexts [43, 44, 51]. Finally, authors were concerned about the possibility of social manipulation and the internalisation of negative stereotypes [25, 30, 39, 41, 45, 51, 58, 67]. A relational approach emphasises the social constitution of the self and this option may influence how one deals with these potential problems.

Attempts to implement relational autonomy in clinical settings seemed to have difficulties with regard to certain end-of-life care practices. Confidentiality problems and information disclosure issues were repeatedly mentioned [26, 32, 38, 51, 55, 59]. The lack of time in busy departments combined with limited staff numbers was pointed out as well [31, 51, 58]. Stressful conditions in many end-of-life situations could negatively affect a family’s ability to participate in decision-making [27, 37, 46, 57]. Besides, some authors were concerned with the emotional demands and wrong expectations towards healthcare workers. Their new roles could extend clinicians’ responsibilities beyond their usual boundaries [31, 51, 55]. Health care professionals will require additional skills of effective communication and social dynamics [26, 27, 54, 58, 67, 73].

## Discussion

The purpose of this systematic review was to analyse the meaning, foundations, and use of the concept of relational autonomy, as described in end-of-life ethics literature. We analysed relevant articles in medical, philosophy, and theology fields. Main findings are highlighted and considered in depth, with the aim of reaching a clearer path towards better decision-making in healthcare.

### Negative conceptualisation of relational autonomy

In many of the included publications, exploration of the concept of relational autonomy was more ‘a reaction against’ individual autonomy than a positive development of the concept itself. In some articles, we noted a problematic tendency towards oppositional thinking. As presented in the first part of the Results sections, many authors started by presenting a sometimes-too-simplistic portrayal of individualistic autonomy and, only then, did they develop a relational conceptualisation of autonomy. In fact, the latter was conceptualised as a ‘mirror’ of individualistic autonomy. For example, six articles presented tables with opposing/binary characteristics of individual and relational notions [27, 37, 45, 49, 54, 67], and in 17 others, a strongly contrasting way of argumentation was employed [26, 29, 31, 35, 38, 43, 44, 46, 50, 51, 53, 59, 60, 62, 63, 65, 71]. This resulted mostly in an impoverished conceptualisation of relational autonomy, since an antithetical conceptualisation can always be presented without any deliberation or analysis.

A dichotomic approach to conceptualising autonomy overlooks the complexity of the contrasting stance. After all, no position would recognise itself in the caricature depicted by the opponent. By way of illustration, Ikonomidis and Singer’s article is an example of a nuanced understanding of individual autonomy from a liberal perspective, far removed from the portrayal depicted in relational critiques [41]. Clearly enough, this oppositional thinking makes dialogue rather unfruitful.

For the aforementioned reasons, we advocate further development of the concept of autonomy from a stance that simultaneously considers both relational and individual dimensions. Thus, in conceptualising human beings, tension is maintained: no relationality without individuals; no individuality without relations. This points directly to philosophical foundations that can ground an integral concept of autonomy that bridges oppositional tendencies. Relational autonomy may lack what van Heijst calls a ‘strong anthropology’, capable of providing normativity to ethics derived from it [76]. As this very critique is also formulated for care ethics [77], one may identify the need for strong anthropological foundations in order to make relational autonomy a normative concept that can be used in ethical argumentation regarding end-of-life issues. Past authors have taken this approach in their writings.

Joseph Selling, for example, introduces a worthy relational turn in Leuven’s personalism [78]. Louis Janssens, the founder of this tradition, had previously distinguished eight dimensions of the human person [79]. Drawing upon this classical work, Selling thoughtfully changes the order of presentation of these eight dimensions: he starts from relationality to end up at the uniqueness of the human person. By doing so, he gives primary weight to relationality while keeping individuality in a crucial position. From our point of view, Selling’s development of Leuven’s personalism can solve both pitfalls observed in relational approaches to conceptualising autonomy. On the one hand, he avoids dichotomic thinking. Selling’s thinking succeeds in bringing together relationality and individuality into a hermeneutic circle, the concepts not diminishing each other but instead strengthening each other. On the other hand, Selling’s description of the eight dimensions of human persons can be considered a ‘strong anthropology’, capable of providing normativity to ethics derived from it.

### Relational autonomy as a multi-source concept

Analysis of the included articles revealed great variety within relational autonomy interpretations. Many ethical approaches that used the concept of relational autonomy could be identified, namely feminist ethics, care ethics, ethical multiculturalism, phenomenology, personalist ethics, relational ethics, virtue ethics, and different forms of political-philosophical approaches, such as communitarianism, liberalism, etc. Nevertheless, most of the included publications referred to a single source of inspiration and presented a one-sided interpretation. They drew from a narrow range of ethical approaches, in contrast to the variety of ones we identified overall in our review. Therefore, this review might preclude reductionist interpretations of relational autonomy, and by extension, any kind of homogenising or simplifying categorisation of this rich concept.

Relational autonomy is an ethical concept that links to a variety of ethical approaches and therefore cannot be exclusively characterised by a single approach. This finding opens up possibilities for frank dialogue among different approaches. Fox and Swazey, among others, also warn about the effects of increasing polarisation in contemporary ethical debate [80]. Our findings that relational autonomy is a multi-source concept suggest that respect for diversity needs to be elevated in our pluralistic world. Thus, as with our call to reject dichotomic thinking, a well-founded relational approach to conceptualising autonomy can also find common ground in traditional and in, what may initially appear to be, divergent approaches. Exploring these convergencies may facilitate dialogue between secular and religious-based ethics, between Western and non-Western philosophies, and other less-obviously connected fields that might speak to more fully understanding autonomy.

### Operationalisation of relational autonomy

Our review has revealed some distance between theoretical approaches to relational autonomy and its operationalisation in end-of-life practices. As it is sharply stated by Dove and colleagues, there is ‘a curious contrast between the rich array of theoretical critique of individualistic notions of autonomy and the paucity of alternative forms of autonomy in practice’ [81]. A thorough consideration of our results uncovers some potential clues about how to translate theory into practice regarding end-of-life decisions. What relational theorists repeatedly demand is a dialogical stand regarding the whole process of decision-making. Even if the way to implement this dialogical stand varies from one author to another, the practical proposal that reached broader consensus was shared decision-making.

Shared decision-making has been increasingly advocated as an ideal model of decision-making since it was first coined by Veatch [82], and then developed by influential authors such as Levine et al. [83], and Linda and Ezekiel Emanuel [84]. In a historical review published in 2015, Stiggelbout and colleagues show how differing philosophies have contributed to the development and expansion of shared decision-making [85]. Nevertheless, other authors, such as Charles and colleagues, lament that shared decision-making has been ‘rather poorly and loosely’ conceptualised [86]. Reacting to this appeal, Grignoli et al. proposed that relational autonomy should form the philosophical foundation for shared decision-making [27].

On the basis of these three critical comments about the current literature on relational autonomy in end-of-life-care ethics, we formulate some implications for future research. First, we suggest that relational autonomy has to be developed further as a normative concept by identifying strong anthropological foundations and by rejecting antithetical thinking about an individualistic interpretation of autonomy. Second, we advocate engaging in fruitful dialogue between different and complementary approaches to the interpretation of relational autonomy. Third, we point out the need for a practical translation of the concept of relational autonomy into ethical decision-making approaches.

### Strengths and limitations

To the best of our knowledge, this is the first systematic review of argument-based literature concerning relational autonomy in end-of-life care ethics. Its methodological strength comes from the use of validated standards, namely PRESS and PRISMA protocols. All the processes, from the search strategy to the data extraction, analysis, and reporting, followed reproducible procedures that were explicitly noted. Even though we considered only journal articles, but excluded, for example, book chapters, we assume that we have captured the main ethical arguments, since our results were highly saturated. We highlight the fact that our search covered five languages and, therefore, reached a considerable diversity of ethical and cultural traditions. We acknowledge the potential bias of using only English search terms. Nevertheless, our results were derived from studies conducted in various countries of different continents, indicating they have wide geographic representation. Finally, the majority of publications are recent, which allowed us to infer that interest in the topic is growing and to expect new studies to come.

## Conclusion

Many articles have been published on the subject of relational autonomy, but none have focussed on bringing together diverse views on its meaning and how it is used in end-of-life care. Systematically evaluating relevant literature in the health sciences, philosophy, and theology, we identified three themes: first, concepts of relational autonomy tend to be more a ‘reaction against’ an individualistic interpretation of autonomy rather than a positive concept itself; second, relational autonomy is a rich and complex concept, formulated in complementary ways from diverse philosophical sources; third, there is a need for dialogical developments in decision-making in end-of-life situations. Completing this analysis was important, because it may illuminate a path towards better decision-making in end-of-life care healthcare. Further analysis is required, however, to reach consensus on how to develop a structured and standardised decision-making process. Clinicians and ethicists should make it a priority to arrive at an interpretation that translates a consensus concept of relational autonomy into ethically sound decision-making approaches.

## Supplementary information

**Additional file 1.** Example of an individual conceptual scheme.

## Data Availability

Not applicable.

## Abbreviations

PRESS: Peer Review of Electronic Search Strategies
PRISMA: Preferred Reporting Items for Systematic Reviews and Meta-Analyses
QUAGOL: Qualitative Analysis Guide of Leuven

## References

[1] United States. National Commission for the Protection of Human Subjects of Biomedical and Behavioral Research. The Belmont Report: Ethical Principles and Guidelines for the Protection of Human Subjects of Research. Bethesda: The Commission; 1978. 25951677

[2] Beauchamp TL, Childress JF (2013). Principles of biomedical ethics.

[3] Mackenzie C, Stoljar N. Relational autonomy: feminist perspectives on autonomy, agency, and the social self. New York: Oxford University Press; 2000.

[4] O'Neill O. Autonomy and trust in bioethics. Cambridge: Cambridge University Press; 2002.

[5] Oshana M (2016). Personal autonomy in society.

[6] Shih P, Rapport F, Hogden A, Bierbaum M, Hsu J, Boyages J (2018). Relational autonomy in breast diseases care: a qualitative study of contextual and social conditions of patients’ capacity for decision-making. BMC Health Serv Res.

[7] Broom A, Kirby E (2013). The end of life and the family: hospice patients’ views on dying as relational. Sociol Health Illn.

[8] MacDonald H (2007). Relational ethics and advocacy in nursing: literature review. J Adv Nurs.

[9] Nolan MT, Hughes M, Narendra DP, Sood JR, Terry PB, Astrow AB (2005). When patients lack capacity: the roles that patients with terminal diagnoses would choose for their physicians and loved ones in making medical decisions. J Pain Symptom Manag.

[10] Kon AA (2010). The shared decision-making continuum. JAMA..

[11] Billings JA, Krakauer EL (2011). On patient autonomy and physician responsibility in end-of-life care. Arch Intern Med.

[12] Müller-Engelmann M, Keller H, Donner-Banzhoff N, Krones T (2011). Shared decision making in medicine: the influence of situational treatment factors. Patient Educ Couns.

[13] Walter JK, Hwang J, Fiks AG (2018). Pragmatic strategies for shared decision-making. Pediatrics.

[14] Thoresen L, Lillemoen L (2016). “I just think that we should be informed” a qualitative study of family involvement in advance care planning in nursing homes. BMC Med Ethics.

[15] Robins-Browne K, Hegarty K, Guillmen M, Komesaroff P, Palmer V (2017). The role of relational knowing in advance care planning. J Clin Ethics.

[16] Osinski A, Vreugdenhil G (2018). Cancer patient characteristics related to prognosis in patients with metastatic cancer admitted to intensive care: the importance of advance care planning and shared decision making. J Palliat Med.

[17] Seibel K, Krause F, Becker G (2014). Ärztliche Verantwortung gegenüber Palliativpatienten unter dem neuen Paradigma der Kundenorientierung. Ethik in der Medizin.

[18] McDougall R (2013). Systematic reviews in bioethics: types, challenges, and value. J Med Philos.

[19] Mertz M, Kahrass H, Strech D (2016). Current state of ethics literature synthesis: a systematic review of reviews. BMC Med.

[20] Broeckaert B, Federation TF (2009). Treatment decisions in advanced disease: a conceptual framework. Indian J Palliat Care.

[21] McCullough LB, Coverdale JH, Chervenak FA (2004). Argument-based medical ethics: a formal tool for critically appraising the normative medical ethics literature. Am J Obstet Gynecol.

[22] McGowan J, Sampson M, Salzwedel DM, Cogo E, Foerster V, Lefebvre C (2016). PRESS peer review of electronic search strategies: 2015 guideline statement. J Clin Epidemiol.

[23] Liberati A, Altman D, Tetzlaff J, Mulrow C, Gøtzsche PC, Ioannidis JPA (2009). The PRISMA statement for reporting systematic reviews and meta-analyses of studies that evaluate healthcare interventions: explanation and elaboration. BMJ..

[24] Dierckx de Casterlé B, Gastmans C, Bryon E, Denier Y (2012). QUAGOL: a guide for qualitative data analysis. Int J Nurs Stud.

[25] Ho A (2008). Relational autonomy or undue pressure? Family’s role in medical decision-making. Scand J Caring Sci.

[26] Walker P, Lovat T (2015). Concepts of personhood and autonomy as they apply to end-of-life decisions in intensive care. Med Health Care Philos.

[27] Grignoli N, Di Bernardo V, Malacrida R (2018). New perspectives on substituted relational autonomy for shared decision-making in critical care. Crit Care.

[28] Głos A (2016). Solidarity in healthcare–the challenge of dementia. Diametros..

[29] Prainsack B (2018). The “we” in the “me” solidarity and health care in the era of personalized medicine. Sci Technol Hum Values.

[30] Ho A (2008). The individualist model of autonomy and the challenge of disability. J Bioethical Inquir.

[31] Wardrope A (2014). Authenticity and autonomy in deep-brain stimulation. J Med Ethics.

[32] Taboada P, Bruera E (2001). Ethical decision-making on communication in palliative cancer care: a personalist approach. Support Care Cancer.

[33] Wilson F, Ingleton C, Gott M, Gardiner C (2014). Autonomy and choice in palliative care: time for a new model?. J Adv Nurs.

[34] Martínez Gómez JA (2010). La bioética y los enfoques del final de la Vida. Revista Cubana de Salud Pública.

[35] Brauer S (2008). Die Autonomiekonzeption in Patientenverfügungen–die Rolle von Persönlichkeit und sozialen Beziehungen. Ethik in der Medizin.

[36] Dudzinski DM, Shannon SE (2006). Competent patients’ refusal of nursing care. Nurs Ethics.

[37] Chan HM (2004). Sharing death and dying: advance directives, autonomy and the family. Bioethics..

[38] Tan Kiak Min M (2017). Beyond a Western bioethics in Asia and its implication on autonomy. New Bioethics.

[39] Mackenzie C, Rogers W (2013). Autonomy, vulnerability and capacity: a philosophical appraisal of the mental capacity act. Int J Law Context.

[40] Marx G, Boakye SO, Jung A, Nauck F (2014). Trust and autonomy in end of life: considering the interrelation between patients and their relatives. Curr Opin Support Palliat Care.

[41] Ikonomidis S, Singer PA (1999). Autonomy, liberalism and advance care planning. J Med Ethics.

[42] Gastmans C, Naulaers G, Vanhole C, Denier Y (2013). From birth to death? A personalist approach to end-of-life Care of Severely ill newborns. Christian Bioethics: Non-Ecumen Stud Med Moral.

[43] Krishna LK, Te Tay J, Watkinson DS, Yee AC (2015). Advancing a welfare-based model in medical decision. Asian Bioethics Rev.

[44] Krishna LK, Watkinson DS, Beng NL (2015). Limits to relational autonomy—the Singaporean experience. Nurs Ethics.

[45] Walter JK, Ross LF (2014). Relational autonomy: moving beyond the limits of isolated individualism. Pediatrics..

[46] Blackler L (2016). Compromised autonomy: when families pressure patients to change their wishes. J Hosp Palliat Nurs.

[47] Whelton BJ (2008). Human nature: a foundation for palliative care. Nurs Philos.

[48] Bosisio F, Jox RJ, Jones L, Rubli TE. Planning ahead with dementia: what role can advance care planning play? A review on opportunities and challenges. Swiss Med Wkly. 2018;148:w14706. 10.4414/smw.2018.14706 30594990

[49] Schicktanz S, Schweda M (2012). The diversity of responsibility: the value of explication and pluralization. Medicine Studies.

[50] Breslin JM (2005). Autonomy and the role of the family in making decisions at the end of life. J Clin Ethics.

[51] Gilbar R, Miola J (2014). One size fits all? On patient autonomy, medical decision-making, and the impact of culture. Med Law Rev.

[52] Lolich L, Lynch K (2017). No choice without care: palliative care as a relational matter, the case of Ireland. Soundings: Interdisciplin J.

[53] Wright MS (2017). End of life and autonomy: the case for relational nudges in end-of-life decision-making law and policy. Maryland Law Rev.

[54] Wallner J (2010). Organisation medizinischer Entscheidungen am Lebensende. Intensivmedizin und Notfallmedizin.

[55] Donchin A (2000). Autonomy, interdependence, and assisted suicide: respecting boundaries/crossing lines. Bioethics..

[56] Schotsmans P, Gastmans C (2009). How to deal with euthanasia requests: a palliative filter procedure. Camb Q Healthc Ethics.

[57] Rosenberg T, Speice J (2013). Integrating care when the end is near: ethical dilemmas in end-of-life care. Fam Syst Health.

[58] van Wijngaarden E, Goossensen A, Leget C (2018). The social–political challenges behind the wish to die in older people who consider their lives to be completed and no longer worth living. J Eur Soc Policy.

[59] Candib LM (2002). Truth telling and advance planning at the end of life: problems with autonomy in a multicultural world. Fam Syst Health.

[60] Mona M (2008). Wille oder Indiz für mutmaßlichen Willen?. Ethik in der Medizin.

[61] Gaille M, Horn R (2016). The role of ‘accompagnement’in the end-of-life debate in France: from solidarity to autonomy. Theor Med Bioeth.

[62] Shildrick M (2008). Deciding on death: conventions and contestations in the context of disability. J Bioethical Inquir.

[63] Borgstrom E, Walter T (2015). Choice and compassion at the end of life: a critical analysis of recent English policy discourse. Soc Sci Med.

[64] Krystallidou D, Devisch I, Van de Velde D, Pype P (2017). Understanding patient needs without understanding the patient: the need for complementary use of professional interpreters in end-of-life care. Med Health Care Philos.

[65] Rigaux N (2011). Autonomie et démence II: être représenté et autonome: Une combinaison possible?. Ger Psychol Neuropsychiatr Vieil.

[66] Stajduhar K, Funk L, Jakobsson E, Öhlén J (2010). A critical analysis of health promotion and ‘empowerment’ in the context of palliative family care-giving. Nurs Inq.

[67] Baker FX, Gallagher CM (2017). Identifying and managing undue influence from family members in end-of-life decisions for patients with advanced cancer. J Oncol Pract.

[68] Guerrero MV (2014). Aportando valor al cuidado en la etapa final de la cronicidad. Enferm Clin.

[69] Guex P, Bonneterre ME (2006). Problématique du choix et «toxicité» ou effets collatéraux des traitements oncologiques du point de vue psychologique et éthique. Éthique des traitements critiques en oncologie. Oncologie..

[70] Schotsmans PT (2003). Relational responsibility, and not only stewardship. A Roman Catholic view on voluntary euthanasia for dying and non-dying patients. Christ Bioeth.

[71] Siddiqui S (2016). Ethical challenges facing advance care planning. Asian Bioethics Rev.

[72] Wright DK, Gros CP (2012). Theory inspired practice for end-of-life cancer care: an exploration of the McGill model of nursing. Can Oncol Nurs Jl/Revue Canadienne de Soins Infirmiers en Oncologie.

[73] Thiele T, Dunsford J (2017). Nurse leaders’ role in medical assistance in dying: a relational ethics approach. Nurs Ethics.

[74] Tavares CQ (2013). Espiritualidade e bioética: prevenção da “violência” em instituições de saúde. Revista Pistis Praxis.

[75] Tse CY, Chong A, Fok SY (2003). Breaking bad news: a Chinese perspective. Palliat Med.

[76] van Heijst JE (2011). Professional loving care: an ethical view of the health care sector.

[77] Vanlaere L, Gastmans C (2011). A personalist approach to care ethics. Nurs Ethics.

[78] Selling J, Hoose B (1998). The human person. Christian ethics: an introduction.

[79] Janssens L (1980). Artificial insemination: ethical considerations. Louvain Stud.

[80] Fox RC, Swazey JP (2005). Examining American bioethics: its problems and prospects. Camb Q Healthc Ethics.

[81] Dove ES, Kelly SE, Lucivero F, Machirori M, Dheensa S, Prainsack B (2017). Beyond individualism: is there a place for relational autonomy in clinical practice and research?. Clin Ethics.

[82] Veatch RM (1972). Models for ethical medicine in a revolutionary age. Hast Cent Rep.

[83] Levine MN, Gafni A, Markham B, MacFarlane D (1992). A bedside decision instrument to elicit a patient's preference concerning adjuvant chemotherapy for breast cancer. Ann Intern Med.

[84] Emanuel EJ, Emanuel LL (1992). Four models of the physician-patient relationship. JAMA..

[85] Stiggelbout AM, Pieterse AH, De Haes JC (2015). Shared decision making: concepts, evidence, and practice. Patient Educ Couns.

[86] Charles C, Gafni A, Whelan T (1997). Shared decision-making in the medical encounter: what does it mean? (or it takes at least two to tango). Soc Sci Med.

