# Supplementary material for: Relational autonomy: what does it mean and how is it used in end-of-life care? A systematic review of argument-based ethics literature
Source: BMC Med Ethics. 2019 Oct 26;20:76. doi: 10.1186/s12910-019-0417-3 (PMC6815421; doi:10.1186/s12910-019-0417-3)
Supplement: Supplementary file 1 — Additional file 1. Example of an individual conceptual scheme. [file 12910_2019_417_MOESM1_ESM.pdf]

**31**	Walter, J. K., and L. F. Ross. "Relational Autonomy: Moving Beyond the Limits of Isolated Individualism." *Pediatrics* 133 Suppl 1 (Feb 2014): S16-23.

- **Meaning**: rich conception of RA (contrast against Individualistic model)
- **Ethical approach**: feminist approach and care ethics
- **Foundation concepts**: "second persons"
- **Anthropological concepts**: embodiment, interconnectedness, self-in-dialogue, dependency,
- **Impact in EOL debate**: shared decision making.
- **Target population**: adolescents and minors.
- **Supporting Authors**: Mackenzie and Stojlar (feminism), Gilligan (Care ethics), B&C (principlism)

Two models of autonomy → lead to different ethical considerations and practices

**INDIVIDUALISTIC MODEL**	→ **RELATIONAL AUTONOMY**
**Origin** in Beauchamp and Childress. It has dominated medical ethics since 1970's. Autonomy is the guiding principle of the doctor-patient relationship.	**Origin** in the 1980's feminist philosophers. It led to the development of an ethics of care. Contemporary claim for a reconceptualization of autonomy.
"**In-control agent**": a self-sufficient, self-interested individual who search maximizing his personal gains. Focus on the individual dimensions of personhood.	**Agents' identities** are formed within the context of social relationships and shaped by a complex of intersecting social determinants. Focus on intersubjective and social dimensions of selfhood and identity. Self-realization is only achieved relationally.
**Self-reliance**: the patient's independency is under constant threat by others' influence (seduction, manipulation)	**Dependency** and **interconnectedness**: persons are socially embedded. We are "second persons" (Baier): essentially heirs to other persons. Our personality is fundamentally and ineradicably revealed and built in relations and responses of others.
**Clinicians** are supposed to provide full information to 'consumers' about their diagnosis, prognosis, and treatment.	**Clinicians** share the responsibility with the parents in searching the best interests of the child. It is a medical obligation to offer recommendations if the family/patient demands it. A clinician cannot abdicate of his responsibility to care for the emotional needs of patient and family.

**Static**, punctual, permanent and synchronic view of identity, interests, and beliefs.	**Dynamic** identity: interests, ends, and beliefs are continually constructed and reconstructed in dialogic processes with other people, as well as with traditions and history.
Clinicians suggestions is a violation of patient's or family's autonomy = **paternalistic** interference.	Valuing the input of others (relatives or physicians) or **engaging** them in important decisions is not an abdication of autonomy.
**Rational** deliberation is privileged: emotions compromise one self-control. Search for objectivity.	The 'self-in-dialogue' is **emotional** and embodied. Role of imagination and emotional dispositions. Ethical significance of trust, intimacy, and empathy.
'**All or nothing'** autonomy: minors and disabled absolutely lack of decision-making capacity.	Promotes the adolescent's **emerging** autonomy. More emphasis on children's capacities.
Autonomy and beneficence (the child's best interests) are the only two **values** at stake.	The interests of the child need to be **balanced** against the interests of the family (problematic example: the 'anointing of the sick' in pg. S21)
**Discussions** should focus on "the **facts**". They are limited to physician and patient (or legal surrogate)	**Discussions** are open to other family members and community participants.<br>Emotional **meetings** are ethically valued.
**Risk** of leaving the patient and family isolated. "You are on your own. You should be able to think clearly about this"	**Risks** of oppressive socialization: unduly influence, lack of development of competencies, capacities, and individual's ability to bring autonomous desires to fruition.
The **final outcome** is an individual **decision**	**Final outcome** is achieved through shared decision-making **process**
